# Exploratory Space–Time Analyses of Reported Lyme Borreliosis Cases in France, 2016–2019

**DOI:** 10.3390/pathogens10040444

**Published:** 2021-04-08

**Authors:** Wen Fu, Camille Bonnet, Julie Figoni, Alexandra Septfons, Raphaëlle Métras

**Affiliations:** 1 Sorbonne Université, INSERM, Institut Pierre Louis d’Épidémiologie et de Santé Publique, F75012 Paris, France; camille.bonnet@iplesp.upmc.fr (C.B.); raphaelle.metras@inserm.fr (R.M.); 2 Santé publique France, F94410 Saint-Maurice, France; Julie.FIGONI@santepubliquefrance.fr (J.F.); Alexandra.SEPTFONS@santepubliquefrance.fr (A.S.)

**Keywords:** Lyme borreliosis, spatial epidemiology, surveillance, *Ixodes ricinus*

## Abstract

In recent decades, the incidence of Lyme borreliosis (LB) in Europe seems to have increased, underpinning a growing public health concern. LB surveillance systems across the continent are heterogeneous, and the spatial and temporal patterns of LB reports have been little documented. In this study, we explored the spatio-temporal patterns of LB cases reported in France from 2016 to 2019, to describe high-risk clusters and generate hypotheses on their occurrence. The space–time *K*-function and the Kulldorf’s scan statistic were implemented separately for each year to evaluate space–time interaction between reported cases and searching clusters. The results show that the main spatial clusters, of radius size up to 97 km, were reported in central and northeastern France each year. In 2017–2019, spatial clusters were also identified in more southern areas (near the Alps and the Mediterranean coast). Spatio-temporal clustering occurred between May and August, over one-month to three-month windows in 2016–2017 and in 2018–2019. A strong spatio-temporal interaction was identified in 2018 within 16 km and seven days, suggesting a potential local and intense pathogen transmission process. Ongoing improved surveillance and accounting for animal hosts, vectors, meteorological factors and human behaviors are keys to further elucidate LB spatio-temporal patterns.

## 1. Introduction

Lyme borreliosis (LB) is the most prevalent tick-borne zoonosis in Europe [1]. This infectious disease is caused by the spirochete bacteria, *Borrelia burgdorferi* sensu lato complex, transmitted to humans through the bite of infectious hard ticks (Family Ixodidae) [2]. Surveillance systems analysis and research designed to study LB in Europe have shown that its incidence seems to have increased in recent decades; although, this trend is not homogeneous [1,3,4,5,6,7]. LB patients usually present a typical rash called erythema migrans (EM), in the first few weeks following infection [2,8,9]. If not present, not recognized or not treated, the bacteria can disseminate to other organs and cause rare but severe manifestations (e.g., arthritic or neurologic disorders) [2,8]. Clinical manifestations of LB may vary with different *Borrelia* genospecies [8,9]. In Europe, more than five species of *Borrelia* can cause human LB (only one species in North America), resulting in various manifestations and potential co-infection in LB patients [8,9]. The three dominant pathogenic species are *Borrelia garinii*, *Borrelia afzelii*, and *Borrelia burgdorferi*, which have been described to be often associated with neuroborreliosis, acrodermatitis chronica atrophicans, and Lyme arthritis, respectively [8,9]. LB surveillance systems across the continent are heterogeneous. Several endemic countries in northern and central Europe have listed LB as a mandatory notifiable disease, whereas in other countries, the collection of LB data is based on sentinel surveillance and hospitalization data [10,11]. Due to the heterogeneity of LB epidemiology and data collection at a continental level, it is difficult to estimate an overall incidence. Nevertheless, field studies are good evidence of the geographical expansion of *Ixodes ricinus* and *Borrelia* in Europe, especially in northern areas and at a higher altitude, which may be related to global warming, landscape alteration, biodiversity change, or a combination of these, increasing public health concerns [12,13,14,15,16].

France is located in western Europe (longitudes 5° W–8° E), which partially overlaps with the zone of high prevalence of *Borrelia* infections in questing nymphal *Ixodes ricinus* (longitudes 5° E–25° E) [17]. Since 2009, LB has been included as one of the health indicators monitored by French general practitioners from the national sentinel network (Réseau Sentinelles) [7]. This network is the source of a real-time epidemiological surveillance system and composed of voluntary general practitioners (hereafter referred to as SGPs, for Sentinelles General Practitioners) across mainland France [18]. Based on the collected LB data, the estimated LB incidence remained stable during 2009–2015, but has increased from 2016 (from 46 (95% CI [34; 58]) in 2009 to 84 (95% CI [70; 98]) per 100,000 inhabitants in 2016) [7]. A spatial heterogeneity of LB incidence between regions (the largest French administrative district, level 1) and years has been reported [7,19,20]. However, very few published studies formally explored the spatial and temporal patterns of LB reports in Europe [21] or in France. Identifying and visualizing diseases clusters can be used to describe the spatial pattern of their occurrence, point out their potential expansion trends, and highlight their high-risk periods requiring special attention [22,23,24,25]. Characterizing the presence of space–time interaction in disease distribution helps to understand the underlying transmission processes and estimate the intensity of infectiousness [26]. It is also useful to assist public health officials in designing targeted preventive measures and control plans. In this study, we examined 916 validated LB cases reported through the Réseau Sentinelles in mainland France from 2016 to 2019 to explore the spatio-temporal patterns of LB cases and compare them across those years. First, we used the space–time *K*-function to characterize the space–time interactions each year and to quantify the excess risks. In a second step, we used a Poisson model based on the Kulldorf’s scan statistic to search for spatial and spatio-temporal clusters at the commune level, describe the detailed information of clusters, and generate hypotheses on cluster occurrence.

## 2. Results

### 2.1. Descriptive Analyses

**Figure 1:**
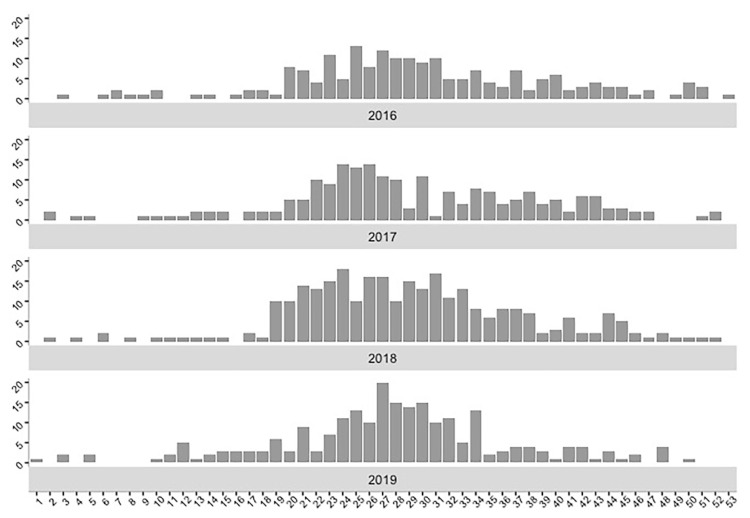
Number of weekly reported Lyme borreliosis (LB) cases in mainland France, from 2016 to 2019.

The number of reported LB cases included each year was 194 cases in 2016, 204 cases in 2017, 288 cases in 2018, and 230 cases in 2019. Figure 1 shows the number of LB cases reported per week and per year. We noticed that LB cases can occur at any time of the year, but very few cases were reported between December and February (weeks 1 to 8 and weeks 50 to 53). During 2016–2019, most LB cases emerged between May and August (71%, *n* = 650 cases) from week 21 to week 34.

**Figure 2:**
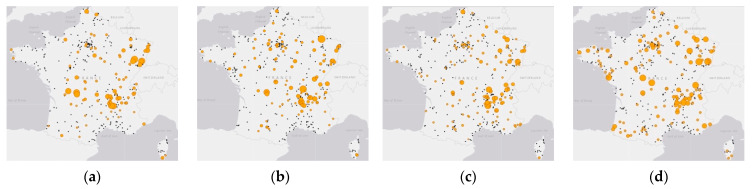
Geographical distribution of communes with active Sentinelles General Practitioners (SGPs) and total number of LB cases reported per commune in mainland France in 2016 (**a**), 2017 (**b**), 2018 (**c**), and 2019 (**d**). Black dots indicate communes under surveillance that reported no LB cases in a year, and yellow circles indicate communes that reported LB cases. The size of the circle is proportional to the number of cases and ranges from 1 to 14 cases. Map Source: ESRI world light gray base map from the TMAP library in the statistical package R version 3.6.2 [27].

Regarding spatial distribution, Figure 2a–d show the geographical distribution of communes with active SGPs and the annual number of LB cases reported in each of these communes. When the location of the tick-bite was available and discordant with the location of the reporting commune, we adjusted these cases accounting for the coordinates of the tick-bite location (See Methods). This corresponded to 11 cases in 2016 (5.7%) from 11 departments, 14 cases in 2017 (6.8%) from 13 departments, 19 cases in 2018 (6.6%) from 17 departments, and 20 cases in 2019 (8.7%) from 18 departments. Furthermore, over the four years of study, the number of communes with active SGPs increased (371, 391, 414, and 467, in 2016–2019), as well as the number of communes reporting LB cases (91, 107, 129, and 130) (Figure 2a–d). It seemed that most cases were gathered in northeastern and southeastern France each year. Compared with the previous three years (2016–2018), we observed an increase in new LB cases in 2019 distributed in Brittany and along the Pyrenees in northwestern and southwestern France, which may be related to the increase of communes with active SGPs in these areas (Figure 2d).

### 2.2. Space–Time K-Function Analysis

Table 1 lists the spatial extent and time period of space–time interactions each year and the corresponding *p*-values. The value of $D_{0}\left(s,t\right)$ was used to evaluate the excess risk attributed to space–time interactions and estimated from 0 to 3.28, expressed as a function of time (*x*-axis, in seven-day increments), and spatial distance (*y*-axis, in 2 km increments). A statistically significant strong space–time interaction was detected in 2018 only (*p*-value = 0.02). Detailed examinations of the $D_{0}\left(s,t\right)$ values in 2018 showed that within 16 km and within one week, the excess risk, $D_{0}\left(s,t\right)$ value was above two. This suggests that, on average, the cumulative number of LB cases observed within a 16-km radius centered on a given LB case and within the week following the reporting date of the given case was at least three times the number expected under the hypothesis of the absence of space–time interaction. Then, the intensity of the space–time interaction decreased from 2 to unity within a week and reached 34 km (excess risk, 1 < $D_{0}\left(s,t\right)$ < 2), indicating that the cumulative number of LB cases observed within a 34 km radius circle centered on the given case exceeded at least twice the expected number, still under the hypothesis of the absence of space–time interaction.

### 2.3. Cluster Analysis

#### 2.3.1. Spatial Clustering Detection

**Figure 3:**
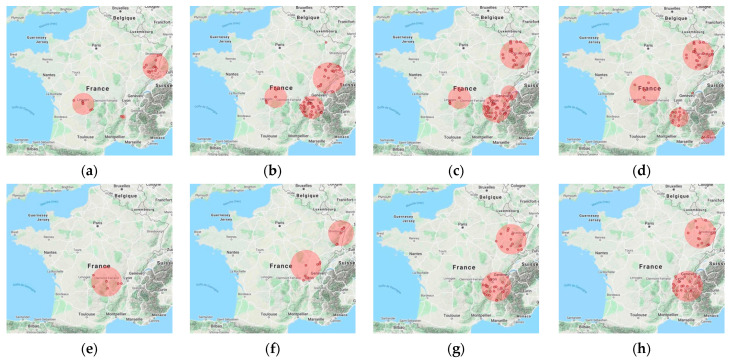
Spatial clusters detected in 2016 (**a**), 2017 (**b**), 2018 (**c**), 2019 (**d**) and spatio-temporal clusters detected in 2016 (**e**), 2017 (**f**), 2018 (**g**), 2019 (**h**). Red shaded circles indicate the geographical scope of clusters, and red dots indicate the high-risk communes with active SGPs. Detailed information is provided in Table 2 and Table 3. Map source: Google maps combined in SaTScan version 9.6.1 [28].

A total of 16 significant spatial clusters were detected (3 in 2016, 4 in 2017, 4 in 2018, and 5 in 2019), mainly located in the same regions each year (Grand Est (GE), Nouvelle Aquitaine (NA), and Auvergne-Rhône-Alpes (ARA)), with a maximum radius up to 97.3 km. Clusters are mapped in Figure 3a–d and detailed in Table 2. In NA region, clusters were reported at similar locations in the four years, with a radius at 69 km in 2016–2018 and expanding to 95.9 km in 2019 (clusters 1, 6, 11, and 14). In GE region, a cluster was found in Alsace in 2016 (cluster 2, radius = 79.3 km). In 2017, it covered Alsace and the adjacent region (Bourgogne-Franche-Comté, BFC), with a maximum spatial radius up to 97.3 km (clusters 7). In the following years (2018 and 2019), two similar clusters were described in GE region (Alsace and Lorraine), exhibiting a slight increase in radius from 87.6 km to 92.5 km (cluster 9 and cluster 12). In ARA region, a two-commune cluster was found in 2016 with a radius of 14.5 km (cluster 3). Larger ones were also found in 2017–2019, with a radius reaching 88.3 km in 2018 (clusters 4, 8, and 13). Finally, a two-commune cluster was reported in 2019 in the Alpes-Maritimes department in the southeastern part of Provence-Alpes-Côte d’Azur (PACA) region (cluster 16).

#### 2.3.2. Space–Time Clustering Detection

Seven significant spatio-temporal clusters were detected (1 in 2016, 2 in 2017, 2018, and 2019), overlapping with spatial clusters in ARA and GE regions (Figure 3e–h, Table 3). In 2016, a cluster was described in central France (early July to early August, one month) between NA and ARA regions (cluster 17). In 2017, one cluster was reported in the northeastern part of the cluster 17 and appeared earlier in the year (cluster 18, late May to late June, one month). In early July of the same year, another cluster was also observed in GE region (Alsace), bordering Germany, and lasting two weeks (cluster 19). In 2018–2019, larger space–time clusters were reported in the same area, with an increase in space of 15 km and in time window over two months (Clusters 21 and 22). In addition, in the eastern part of ARA region, near the Alps, two similar clusters were respectively described (late May to late August, 3 months), with a maximum spatial range reaching 97.7 km (cluster 20 and cluster 23). 

## 3. Discussion

This paper presents the first study exploring the spatio-temporal patterns of LB occurrence in mainland France, using LB surveillance data collected by the Réseau Sentinelles. The space–time *K*-function has been widely used in public health research to investigate the spatio-temporal interaction of emerging infectious diseases [26,29,30,31]. In our case, *Borrelia* is transmitted to humans by an infectious tick bite. Thus, the identified space–time interaction can be interpreted as reflecting the underlying transmission of *Borrelia* at the interface between animals, ticks, and humans. The clusters identified during the study period were mainly located in the northeastern, southeastern, and central parts of France, showing continuity characteristics between the years. The maximum spatial window was up to 97 km, and the maximum temporal window lasted for three months (May to August).

Our results show that there was a significant short-term spatio–temporal interaction in 2018, not identified in the other years and suggesting the presence of a local, short, and intense pathogen transmission process. This can be explained by several hypotheses. It is known that the three tick life stages (larvae, nymph, and adult) require a blood meal before entering to the next stage [2]. Small rodents are one of the important hosts of larval ticks, and also, are often considered to be the main reservoirs of *B. afzelii* and *B. burgdorferi* [32]. They are not long-distance migratory animals. The larval ticks can be infected with bacteria *Borrelia* during blood feeding before molting into nymphs [33]. Therefore, we supposed that the distribution of infected nymphal ticks may be localized but scattered in different areas. Humans are incidental hosts and LB in human is mostly caused by the bite of infected nymphs [34]. It is possible that a high concentration of infected animals (e.g., mice, hares, and chipmunks) over a short period of time has led to an increase in infected nymphal ticks [33], which, coupled with the overlap of human outdoor recreation sites, resulted in more LB patients presenting to the SGPs. The aggregation of a large number of case reports in a short time period of time may result in the detection of an important spatiotemporal interaction process [26]. In 2018, we noticed that many cases were reported between mid-July and mid-August in several neighboring communes in the ARA region including 13 LB cases reported in one of them. In contrast, we did not observe a similar situation in 2016, 2017, and 2019. There may exist spatial or temporal proximity in the case distribution, but not present simultaneously (in space and in time) during these three years. In addition, the average weekly participation level in monitoring activity was almost the same for the SGPs during 2017 and 2018, but higher in 2019 [35]. This suggests that the spatio–temporal interactions we observed in 2018 were not necessarily caused by an increase or change in SGPs’ reporting activities, but may be related to many other factors, such as specific meteorological conditions, changes in local ecosystems, and higher attendance of individuals. For example, a Swedish study showed that a mild temperature summer and a relative humidity above 86% may increase the host-seeking activities of nymphal ticks *Ixodes ricinus*, which could be one factor associated with the increased incidence of LB in their country during the year [36].

The high-risk clusters detected in our analysis are consistent with the regions that already reported high incidence of LB in France [7,20,35]. The estimated high-risk time window was concentrated between May and August, which could be explained by the seasonal characteristics of tick host-seeking behavior, coupled with human outdoors activities [33]. This seasonality has also been shown in numerous epidemiological studies [6,7,36,37,38]. In the purely spatial cluster analysis, the scanning temporal window covers a whole year (i.e., no restriction in the time dimension, to avoid the preselection bias), making the spatial window closer to the cluster’s size evaluation [24]. In this sense, we notice a large range in the size of spatial clusters, from a single commune to 48 communes. Our results can complement the descriptive research on the incidence of LB between regions [7] and point out potential high-risk areas within those regions. The scanning statistics approach used enables the detection of disease clusters without the constraints of administrative boundaries [24], and also informs the further selection of potentially finer geographical resolutions to investigate the biotic and abiotic factors related to LB distribution in mainland France.

From 2017, spatial clusters were also reported in southeastern France and distributed along the Alps. A field study conducted in the western Alps during the same period demonstrated an expansion of geographical distribution of *Ixodes ricinus*, which could be related to meteorological change (e.g., increased mean winter temperatures) and a high presence of red deer in alpine regions [13]. In Europe, deer is considered to be an important reproductive host for female adult ticks [8,33]. The abundance of deer could also be one of the explanations for the cluster found in Limousin (NA) in central France, where the deciduous forest and pasture constitute an ideal living environment for ticks and animal hosts [39]. Further data on tick abundance, reservoirs and tick bite locations [40] will help in further investigating the spatial pattern of LB occurrence.

In 2019, we detected a spatial cluster which consisted of two communes in the Alpes-Maritimes (PACA). It has been suggested that the hot and dry summer climate, that we find in the Mediterranean area, would make it more difficult for *Ixodes ricinus* to develop [41]. In particular, the characteristics of wilt leaves in the forest are difficult to maintain a high humidity microenvironment suitable for the survival of *Ixodes ricinus* and their questing activities [41]. However, it was confirmed that the tick bites of these reported LB cases occurred within the cluster described (not outside the cluster), with patients reporting to have been bitten in the Alpes-Maritimes department. The SGPs reporting those cases participated in the four-year surveillance activities and had a relatively regular reporting frequency. We have several assumptions for this observed cluster. First, we notice that the location of cases reported were close to natural parks. The mixed landscape of residential and forested lands provides favorable conditions at the interface between habitat for the tick reservoirs, and presence of humans, which may increase the risk of human exposure to tick bite [33,34]. Second, an increase or a change in the hosts community size or composition may lead to a higher infection rates in questing nymphal ticks [42]. Assuming that the tick density remains unchanged, more infected ticks will also increase the risk of LB infection in local residents. Third, this cluster is a purely spatial test, with a *p*-value much higher than the other detected clusters in GE and ARA (0.045 vs. < 0.001). The reporting date of cases spans a wide range, from March to October (at most one case per month), and we did not detect the spatio-temporal cluster in the same area. Further surveillance is necessary to confirm this trend.

Our findings have several limitations. First, the surveillance data used represent only a fraction of LB cases in France and may not be fully representative. However, the case definition used and procedure to report give weight to the possible comparison across years. Second, the distribution of LB cases depends on the geographical location of each SGP. In the absence of information on the location of tick exposure, we assumed that the SGP location (commune and department) was the residence of the reported LB patients, i.e., the location where the tick bite occurred, and that the commune centroid was used as the location of the case. In our dataset, tick bite location was available for approximately 50% of cases (accurate to department only), with 84.4–88.8% of the reporting and biting department being similar. Whilst this supports that our assumption remains realistic, one should carefully notice that the reported tick bite by the patient did not necessarily mean that tick bite was the infectious one. Additionally, this coincides with a recent German study investigating 33,153 LB patients which reported that 90.6% of people were bitten at their place of residence [37]. Moreover, a large majority of clusters found in our study (20 out of 23 clusters) covered one to multiple departments, suggesting that the location accuracy at the commune level within the same department had little effect on the clustering results. Furthermore, when the tick bite location was known and different from the reporting department, the commune at the center of the tick bite department was used as the case location. In this scenario, small artificial clusters may have been created if multiple cases claimed to have been bitten by ticks in the same department within a year. In our dataset, a large majority of the adjusted cases originated from different departments each year (as described in the results). In addition, the three small clusters, which consisted of one or two communes (clusters 3, 5, and 15) were not generated by those adjusted cases. Third, the date used was the date when the SGP reported the LB case to the Réseau Sentinelles, for the following reasons: (i) the reporting dates are subject to the unified standard of the national sentinel reporting system and were therefore available for all cases, whilst more than half of cases (53%, *n* = 484) missed the date of tick bite, and 47 cases (5%) did not have a corresponding diagnosis date; (ii) for those cases reporting a tick bite, the median of time interval between the date of tick bite and the reporting date is 14 days (Q1–Q3: 7–25 days), which is consistent with the onset of erythema migrans varying between 2 and 30 days [2]; and (iii) the median of the time between the diagnosis and reporting date is 1 day (Q1–Q3: 0–4 days), which may have little effect on our analyses. Fourth, we assumed that the ratio of GPs to residents at the commune level is equal to the ratio at the departmental level, and that a resident had the same probability of visiting any GP within a commune, which resulted in calculating a population at risk for each commune that does not necessarily reflected the actual situation. Thereby biasing the calculation of LB incidence rate in clusters, it is still proportional to the estimated overall LB incidence and seems unlikely to invalidate the significance of clusters. Last, we removed Corsica from the space–time exploratory analysis because the space–time *K*-function is performed only on a single polygon, and because very few LB cases have been reported in the past four years (four cases in 2016, three cases in 2017, two cases in 2018, and three cases in 2019), precluding a robust use of such statistical tests in that setting.

Our research results help to better understand the spatial and temporal heterogeneity of LB distribution in France. The identified high-risk areas under surveillance may serve as pilots for public health officials to develop more specific LB prevention and control plans. Enhanced LB-specific training of GPs may be need in communes with increased reported cases. Complementing and refining the location of tick bites in data collection is important to understand the overlap patterns between tick habitat and human recreational areas within France. Encouraging more GPs to participate in monitoring activities and expanding the coverage of active SGPs will help to improve LB surveillance nationwide. In addition, there is an increasing need to combine biological (such as, vector, reservoir, and host animals) and meteorological factors and human case surveillance data to predict the occurrence of LB and to further investigate potential risk factors for the clusters identified in this study.

## 4. Materials and Methods

### 4.1. Data Collation and Management

LB data were obtained from the nationwide surveillance of the Réseau Sentinelles in mainland France. Each SGP participating in continuous surveillance activities uses an online system (https://www.sentiweb.fr) to report new LB cases to the Réseau Sentinelles. Case information was collected by the SGP during the medical consultation using a standardized questionnaire [7,19,20]. A case was diagnosed by the presence of erythema migrans alone (no size limit) or late manifestations associated with LB confirmed by laboratory [7]. All LB cases reported to the Réseau Sentinelles were validated by an expert group applying a specific clinical case definition based on the European Union Concerted Action on Lyme Borreliosis (EUCALB) [7]. We extracted the following information: the number of active SGPs each year (defined as at least one report in that year, all health indicators monitored confounded), the number of French GPs, the number of LB cases each year, the commune INSEE code of the SGP (i.e., the national index code used to identify different administrative districts in France, with the commune being the smallest administrative division), the reporting date, the diagnosis date and the tick-bite date of each LB case (if available), and when available, the location of the reported tick bite (precise to the department level, French administrative division, level 2). Geographical information was retrieved from the National Institute of Geography and Forestry (IGN) [43]. Demographic data were obtained from the 2017 national census [44].

### 4.2. Descriptive Analyses

The weekly number of LB cases per year was plotted from 2016 to 2019. To describe the spatial distribution of LB cases, a commune-level French administrative map was extracted. We assumed that cases of LB infection occurred in the commune of residence and consulted the local SGP, i.e., the commune of the SGP reporting cases, and we used the commune’s centroids for mapping. However, when the data specified that the tick bite occurred outside the reporting commune, we used that spatial information to adjust the location of the tick bite. For those cases, since the location of the tick bite was only available at the departmental level, we used the centroid of the commune located in the center of that department. All geographical data were projected using the “Réseau Géodésique Français 1993” projection (RGF93, EPSG:7042) and processed using the TMAP library (ESRI World Light Gray base map as a layer) from the statistical package R version 3.6.2 [27].

### 4.3. Space–Time K-Function Analysis

The space–time *K*-function was first used to investigate the space–time interaction among LB cases each year (Supplementary Material S1) [26,45,46]. The study area was mainland France except Corsica Island. The unit of analysis was defined by a LB case with the GPS coordinate (converted to Cartesian units, km) of the centroid of the reporting commune, and the reporting date (converted to day number in a year). We chose a maximum distance of 50 km and 90 days for testing. A *p*-value below 0.05 obtained from 999 Monte Carlo simulations was used to verify that space–time interaction did not happen by chance [46]. The analysis was implemented using the SPLANCS library from the statistical package R version 3.6.2 [27].

### 4.4. Cluster Analyses

We applied the Poisson model based on the Kulldorf’ scan statistic to search for high-risk LB reporting areas and high-risk reporting time periods (i.e., spatial clustering and space–time clustering) in mainland France. We assumed that individuals within a commune are equally likely to visit any GP in the commune (SGPs accounts for approximately 2% of all GP [20]). Therefore, we calculated the adjusted at-risk population, *P_c_’*, in each commune by the number of SGPs and GPs according to the Equations (1) and (2),

(1) $$P_{c}^{\prime }=P_{c}÷N_{GPC}\times N_{SGP}$$

and

(2) $$N_{GPC}=P_{C}÷P_{D}\times N_{GPD}$$

Here $P_{c}$ is the number of inhabitants in commune *c*, $N_{GPC}$ is the total number of GPs in commune *c*, and $N_{SGP}$ is the number of SGPs in commune *c*. Since we only obtained information on the number of GPs at the departmental level (noted as $N_{GPD}$), we assumed that the ratio of the number of departmental-level GPs to the total number of inhabitants in department (referred to as $P_{D}$) is the same as the commune-level, and thus the number of $N_{GPC}$ was estimated by Equation (2).

The centroid of the commune was used as the unit of analysis. In the purely spatial scan statistic, we included all communes with active SGPs. The spatial scan statistic was defined by a circular window centered on the centroids of one of any possible studied communes, with a radius varying from 0 to 100 km (the maximum window size set) [47]. In contrast, in the space–time scan statistic, we only included communes with LB cases, and the reporting date was used as a reference to add a temporal dimension based on spatial scan windows. The formed scan cylinder then moved in space randomly, with a height reflecting any time interval between 14 days and 90 days within the study year. Under the null hypothesis of no clustering, the risk of LB cases being reported should be constant in space (and in time) and proportional to the population size, i.e., the number of observed LB cases within the scan windows is similar to the expected number [48].

For each candidate cluster, the radius (in km), time window size (in dates and days, only for spatio-temporal clusters), the number of communes, the population size, the number of observed and expected LB cases, the relative risk (RR), and the log-likelihood ratio were estimated and reported. The statistical significance of all identified spatial and spatio-temporal clusters was evaluated using 999 Monte Carlo simulations, and with a *p*-value threshold at 0.05. We retained only the significant non-overlapping clusters determined by the Gini coefficient [49]. The most likely cluster was defined by the maximum log-likelihood ratio value, and the other clusters were referred to as secondary clusters and ranked sequentially according to their ratio. All statistical analyses were performed in R version 3.6.2, using the RSCAN package [27] and combined with Google maps in SaTScan version 9.6.1 [28].

## 5. Conclusions

In conclusion, our study explored the spatial and temporal patterns of LB case reports in France over a four-year period from 2016 to 2019. The results show that major spatial clusters were identified in central and northeastern France each year. In 2017–2019, spatial clusters were also found in more southern areas. Spatio-temporal clusters were reported each year between May and August. A strong and significant space–time interaction was identified in 2018, suggesting potentially localized and dense pathogen transmission processes. Ongoing improved surveillance and accounting for animal hosts, vectors, meteorological factors, and human behavior are key to explain the high-risk clusters identified in our study. A spatial model that combines all these factors to further predict LB occurrence in mainland France is needed.

## Figures and Tables

**Table 1:** Results of the space–time *K*-function. Excess risk attributed to the space–time interactions $D_{0}\left(s,t\right)$, and corresponding *p*-values.

Year	Time (90 Days)	Space (50 km)	$D_{0}{\left(s,t\right)}_{\;}$	Upper Time Window	Upper Space Window	*p*-Value
2016	7 days	2 km	>2	7 days	16 km	0.71
			>1	7 days	40 km	
2017	7 days	2 km	>2	na	na	0.18
			>1	7 days	20 km	
2018	7 days	2 km	>2	7 days	16 km	0.02 ^1^
			>1	7 days	34 km	
2019	7 days	2 km	>2	7 days	22 km	0.12
			>1	7 days	40 km	

^1^ significant *p*-value.

**Table 2:** Spatial clusters of reported LB cases detected in France from 2016 to 2019, using the Poisson model based on the Kulldorf’s scan statistic methods (with adjusted at-risk population).

Year/Cluster Number (Region)	RR ^1^	Radius (km)	No. of Communes	Population at Risk	Observed No. of LB Cases	Expected No. of LB Cases	*p*-Value
**2016**							
1. Most likely cluster (NA) ^2^	9.1	69.0	7	7160	25	3.12	<0.001
2. Secondary cluster (GE)	5.7	79.3	14	17,649	37	7.69	<0.001
3. Secondary cluster (ARA)	12.8	14.5	2	3324	17	1.45	<0.001
**2017**							
4. Most likely cluster (ARA)	3.3	73.9	39	49,197	57	21.43	<0.001
5. Secondary cluster (GE)	20.8	0	1	1160	10	0.51	<0.001
6. Secondary cluster (NA-CVL)	6.4	69.2	6	6585	17	2.87	<0.001
7. Secondary cluster (BFC-GE)	3.2	97.3	20	23,811	30	10.37	<0.001
**2018** ^3^							
8. Secondary cluster (ARA)	3.5	88.3	48	60,026	91	33.48	<0.001
9. Secondary cluster (GE)	3.2	87.6	26	29,125	46	16.24	<0.001
10. Secondary cluster (BFC)	5.2	48.5	6	7275	20	4.06	<0.001
11. Secondary cluster (NA-CVL)	3.6	69.9	7	8347	16	4.66	0.02
**2019**							
12. Most likely cluster (GE)	4.0	92.5	28	31,388	41	11.97	<0.001
13. Secondary cluster (ARA)	4.2	62.6	14	16,137	24	6.16	<0.001
14. Secondary cluster (NA-CVL)	5.0	95.9	7	9553	17	3.64	<0.001
15. Secondary cluster (ARA)	10.7	0	1	1508	6	0.58	0.02
16. Secondary cluster (PACA)	9.4	42.2	2	1720	6	0.66	0.045

^1^ RR: the relative risk of a LB case being reported within a spatial window compared to the outside. ^2^ Most likely cluster is defined by the maximum value of the log-likelihood ratio. ^3^ Most likely cluster in 2018 was not a Gini cluster (overlapped with secondary cluster 8) and therefore was not retained. GE = Grand Est; BFC = Bourgogne-Franche-Comté; ARA = Auvergne-Rhône-Alpes; NA = Nouvelle Aquitaine; CVL = Centre-Val de Loire, PACA = Provence-Alpes-Côte d’Azur.

**Table 3:** Spatio-temporal clusters of reported LB cases detected in France from 2016 to 2019, using the Poisson model based on the Kulldorf’ scan statistic methods (with adjusted at-risk population).

Year/Cluster Number (Region)	RR ^2^	Radius (km)	Estimated Time Frame (Days)	Population at Risk (No. of Communes)	Observed No. of LB Cases	Expected No. of LB Cases	*p*-Value
**2016**							
17. Most likely cluster ^1^ (ARA-NA)	10.1	96.5	7 July–10 August (34)	13,566 (7)	11	1.15	0.004
**2017**							
18. Most likely cluster (ARA-BFC)	7.9	96.8	30 May–30 Jun (32)	22,643 (7)	12	1.60	0.01
19. Secondary cluster (GE)	17.1	77.8	28 June–10 July (14)	14,742 (6)	7	0.42	0.02
**2018**							
20. Most likely cluster (ARA)	3.6	96.0	28 May–25 August (89)	106,123 (25)	69	21.57	<0.001
21. Secondary cluster (GE)	3.6	92.6	21 May–14 August (85)	43,935 (15)	31	9.30	0.001
**2019**							
22. Most likely cluster (GE)	3.8	92.5	24 May–09 August (77)	47,063 (15)	29	8.46	0.002
23. Secondary cluster (ARA)	3.4	97.7	5 June–29 August (84)	47,350 (22)	29	9.38	0.01

^1^ Most likely cluster is defined by the maximum value of the log-likelihood ratio. ^2^ RR: the relative risk of a LB case being reported within an estimated spatial and time window compared to that outside the areas and the period. GE = Grand-Est; BFC = Bourgogne-Franche-Comté; ARA = Auvergne-Rhône-Alpes; NA = Nouvelle Aquitaine; CVL = Centre-Val de Loire.

## Data Availability

A special request can be addressed to rs-data@sentiweb.fr.

## Supplementary Materials

The following is available online at https://www.mdpi.com/article/10.3390/pathogens10040444/s1, Details on space–time K-function analysis method.
