# Supplementary material for: Exploratory Space–Time Analyses of Reported Lyme Borreliosis Cases in France, 2016–2019
Source: Pathogens. 2021 Apr 8;10(4):444. doi: 10.3390/pathogens10040444 (PMC8068173; doi:10.3390/pathogens10040444)
Supplement: Supplementary file 1 [file pathogens-10-00444-s001.zip › pathogens-1149710supp.docx]

**Supplementary Material 1**

This supplementary material is hosted by MDPI Pathogens as supporting information alongside the article Exploratory space-time analyses of reported Lyme Borreliosis cases in France, 2016–2019 by Fu et al. on behalf of the authors who remain responsible for the accuracy and appropriateness of the content. The same standards for ethics, copyright, attributions and permissions as for the article apply.

**Space-time *K*-function analysis**

The space-time *K*-function was used to evaluate the spatio-temporal interactions between the LB cases, and this approach is described in detail below.

$K\left( s,t \right)$ is defined as the cumulative expected number of cases within a given time period *t* and within a distance *s* of a previously random chosen LB case, divided by the intensity, defined as the average number of LB cases per unit distance and per unit time [45]. $K\left( s \right)$ and $K\left( t \right)$ are the space *K*-function and the time *K*-function, respectively [45]. Under the null hypothesis of no space-time interaction, $K\left( s,t \right)$ should be equal to the product of $K\left( s \right)$ and $K\left( t \right)$, as described in equation (1).

$K\left( s,t \right)=K\left( s \right)K\left( t \right)$ (1)

If the difference between$K\left( s,t \right)$ and $K\left( s \right)K\left( t \right)$ is great than 0, which means that there exits the space-time interaction between LB cases, denoted as $D\left( s,t \right)$ in equation (2).

$D\left( s,t \right)=K\left( s,t \right)-K\left( s \right)K\left( t \right)>0$ (2)

Since the value of $D\left( s,t \right)$ increases naturally with the prolonging distance and time interval, the ratio $D_{0}\left( s,t \right)$ calculated by equation (3) is often used for interpretation.

$D0\left( s,t \right)=\frac{D\left( s,t \right)}{K\left( s \right)K \left( t \right)}$ (3)

The value of $D_{0}\left( s,t \right)>1$ indicates that the cumulative number of observed cases is at least twice as expected under the null hypothesis of no space-time interaction [26,45]. The higher the value $D_{0}\left( s,t \right)$, the stronger the space-time interaction. 999 Monte Carlo simulations are used to verify that spatio-temporal interactions do not occur by chance, and the reporting date for each case will be randomly reassigned to the case whose location is assumed to be fixed [46]. The null hypothesis is rejected with a *p* value less than 0.05.

26. French, N.P.; McCarthy, H.E.; Diggle, P.J.; Proudman, C.J. Clustering of equine grass sickness cases in the United Kingdom: A study considering the effect of position-dependent reporting on the space-time K-function. *Epidemiol. Infect.* **2005**, *133*, 343–348, doi:10.1017/S0950268804003322.

45. Diggle, P.J. Second-order analysis of space-time clustering. *Stat. Methods Med. Res.* **1995**, 124–136.

46. Besag, J.; Diggle, P.J. *Simple Monte Carlo Tests for Spatial Pattern*; Journal of the Royal Statistical Society. Series C (Applied Statistics), 1977; Volume 26, pp 327-333
